# Discovery and Development of a TGFβ3/IL-13 Plasma Risk Score for Preoperative Discrimination of Benign and Malignant Ovarian Adnexal Masses

**DOI:** 10.3390/diagnostics16152365

**Published:** 2026-07-28

**Authors:** Jesús García-Gómez, Alejandro Bravo-Cuellar, Pablo Cesar Ortiz-Lazareno, Adriana Aguilar-Lemarroy, Luis Felipe Jave-Suárez, Sarah Naomi Guevara-Hernández, Alejandro Bravo-Hernández, Stephanie Mora-López, Raquel Villegas-Pacheco, Johanna Lizeth Sáenz-Santibañez, María Martha Villaseñor-García, Georgina Hernández-Flores

**Affiliations:** 1 Centro de Investigación Biomédica de Occidente (CIBO), División de Inmunología, Instituto Mexicano del Seguro Social (IMSS), Guadalajara 44340, Jalisco, Mexico; jesus.garcia9891@alumnos.udg.mx (J.G.-G.); abravocster@gmail.com (A.B.-C.); pablolazareno@gmail.com (P.C.O.-L.); adriana.aguilar@academicos.udg.mx (A.A.-L.); luis.jave@academicos.udg.mx (L.F.J.-S.); naomiguevarah@gmail.com (S.N.G.-H.); 2 Programa de Doctorado en Ciencias Biomédicas, Centro Universitario de Ciencias de la Salud, Universidad de Guadalajara, Guadalajara 44340, Jalisco, Mexico; 3 Programa de Doctorado en Microbiología Médica, Centro Universitario de Ciencias de la Salud, Universidad de Guadalajara, Guadalajara 44340, Jalisco, Mexico; 4 Servicio de Medicina Interna, Hospital Civil Dr. Antonio González Guevara, IMSS-Bienestar, Tepic 63000, Nayarit, Mexico; alejandrobravohernandez9@gmail.com (A.B.-H.); smol.17@hotmail.es (S.M.-L.); 5 Unidad Académica de Medicina, Universidad Autónoma de Nayarit, Tepic 63000, Nayarit, Mexico; 6 Unidad Médica de Alta Especialidad, Hospital de Ginecología y Obstetricia, Centro Médico Nacional de Occidente, Instituto Mexicano del Seguro Social (IMSS), Guadalajara 44349, Jalisco, Mexico; raquel.villegaspa@imss.gob.mx (R.V.-P.); jlss_2662@hotmail.com (J.L.S.-S.); 7 Centro Universitario de Ciencias Exactas e Ingeniería, Universidad de Guadalajara, Guadalajara 44840, Jalisco, Mexico

**Keywords:** adnexal masses, ovarian cancer, CA125, cytokines, TGF-β, IL-13

## Abstract

**Background/Objectives**: Differentiating between benign and malignant ovarian adnexal masses before surgery is a significant diagnostic challenge. This study examined a wide range of circulating plasma molecules (immune and inflammatory) to identify potential markers to classify tumors as benign or malignant. The goal was to develop a proof-of-concept tool for preoperative risk stratification. **Methods**: Twenty-three plasma molecules were evaluated in women with benign lesions (*n* = 68) and women with malignant ovarian tumors (*n* = 25). To evaluate the behavior of each marker in a normal population, women without adnexal pathology were also included (*n* = 11). The results were compared using the Kruskal–Wallis test with Dunn post hoc pairwise comparisons, the Benjamini–Hochberg correction for multiple comparisons, and ROC analysis. TGF-β isoforms and CA125 were measured in the complete cohort (*n* = 104), while the remaining molecules were assessed in a reduced cohort (*n* = 51). Two classification approaches were developed using TGF-β3 and IL-13: a sequential two-step algorithm and an ordinal logistic regression model. Apparent performance was corrected for optimism using Harrell’s bootstrap procedure. **Results**: Ten molecules reached statistical significance. Among the significant molecules, TGF-β3 identified ovarian lesions regardless of malignant status (AUC = 0.983), while IL-13 showed perfect malignancy-specific separation from benign cases (AUC = 1.000). Their combination in an ordinal logistic regression model produced three non-overlapping diagnostic strata across all three groups, significantly outperforming CA125, HE4, and ROMA at the malignancy-separation boundary (DeLong *p* < 0.05) in our exploratory cohort. **Conclusions**: TGF-β3 and IL-13 represent a biologically complementary marker pair whose combined ordinal regression model shows promising performance and warrants prospective validation in larger, multicenter independent cohorts as a preoperative risk-stratification tool for ovarian adnexal masses.

## 1. Introduction

According to the International Agency for Research on Cancer (IARC), ovarian cancer (OC) was the sixth most common cancer in women worldwide in 2022, with an estimated 324,603 new cases and 206,956 deaths [1]. In Mexico, 5193 new cases were diagnosed in 2022, corresponding to an incidence rate of 6.7 per 100,000 women [2]. OC has a high mortality rate because it is often diagnosed at an advanced stage [3]. High-grade serous carcinoma, the most common histological subtype, arises predominantly from the fallopian tube epithelium [4], accounts for about 70% of cases [5,6], and is associated with a 5-year cause-specific survival rate of 47% [7]. Because clinical outcomes depend strongly on the nature of the lesion at the time of surgery, accurate preoperative risk stratification of ovarian adnexal masses remains a major clinical need. Current assessment relies on CA125, human epididymal protein 4 (HE4), and menopausal status, which together are used to calculate the Risk of Ovarian Malignancy Algorithm (ROMA) [8]. Despite its clinical utility, CA125 has limited sensitivity for early-stage disease, detecting only approximately 50–62% of cases. In addition, elevated CA125 levels are observed in more than 60% of women without ovarian cancer, including those with benign conditions such as endometriosis or pelvic inflammatory disease, as well as those with other malignancies [9]. This limited specificity highlights the need for complementary preoperative biomarkers that better capture the biological complexity underlying adnexal masses.

The tumor microenvironment of OC is characterized by a systemic immune dysregulation involving the reciprocal suppression of Th1-mediated cytotoxic responses and the amplification of Th2-associated immunosuppressive signaling, a pattern that extends beyond the peritoneal cavity and is detectable in the peripheral circulation [10,11]. This systemic immune shift provides a biological rationale for measuring circulating cytokines as candidate markers of the immunological state associated with ovarian pathology in women with adnexal masses.

Among the circulating molecules with established relevance to OC biology, the TGF-β family has been implicated in stromal remodeling, epithelial-to-mesenchymal transition, and immune suppression in the tumor microenvironment [12,13]. Notably, the three TGF-β isoforms are overexpressed at different frequencies in malignant ovarian tumors, TGF-β2 and TGF-β3 more frequently than TGF-β1 [14], and show distinct prognostic associations [15], suggesting that individual isoforms may carry non-redundant biological information. Th2/M2-associated cytokines, including IL-13, IL-5, IL-6, and IL-9, have been identified in the serum of OC patients and linked to tumor progression and immune evasion [10,16,17]. Th1 effector cytokines such as IFN-γ have also been documented in the ovarian tumor microenvironment, where they can paradoxically promote immune evasion [18]. In addition, pleiotropic inflammatory molecules such as TNF-α have been associated with advanced disease stage and poor prognosis through autocrine and paracrine signaling loops [19,20].

Given the biological relevance of these molecular groups to OC pathology and their detectable systemic reflection in the peripheral circulation, the present study evaluated 23 circulating plasma molecules organized into distinct biological groups, including TGF-β isoforms, Th1 effector cytokines, Th2/M2-associated mediators, pleiotropic inflammatory molecules, and CA125 as an established ovarian tumor marker. The latter, with the aim of identifying candidate markers whose plasma concentrations reflect the immunological state associated with tumor presence. Molecules identified in the discovery phase were subsequently used to develop and evaluate two complementary classification approaches, a sequential two-step diagnostic algorithm and an ordinal logistic regression model, as proof-of-concept tools for preoperative discrimination between benign lesions and malignant ovarian adnexal masses.

## 2. Materials and Methods

### 2.1. Study Participants

This study was conducted at the Department of Gynecologic Oncology of the Gynecological Hospital, West Medical Center of the Mexican Institute of Social Security (CMNO, IMSS) in Guadalajara, Jalisco, Mexico. Samples were obtained during the preoperative assessment phase from January 2023 to January 2024, prior to definitive histopathological diagnosis. Ninety-three women with ovarian adnexal masses were recruited alongside 11 control volunteers without known adnexal pathology, whose disease-free status was confirmed by transvaginal ultrasonography and CA125 measurement. Fifteen milliliters of venous blood were drawn from all participants. Plasma samples were collected and stored at −80 °C until use. CA125 concentrations were determined using a two-step chemiluminescent microparticle immunoassay (CMIA) performed on an Alinity immunoassay system with the Alinity i CA 125 II reagent kit (Abbott, Lake Bluff, IL, USA). All data, including pathology data, were obtained from medical records. The exclusion criteria were as follows: (a) age < 18 years, (b) prior preoperative medical intervention, (c) presence of another concurrent malignancy, and (d) missing CA125 tumor marker data. At the time of blood sample collection, no participant had received hormone therapy, radiotherapy, chemotherapy, or other medical interventions. No participant included in the study had evidence of infection, vaginal bleeding, systemic inflammatory disease, or had received antibiotics in the 4 weeks before recruitment. Analyses were performed after histological results were available. All participants provided written informed consent before sample collection, and their data were handled confidentially. No formal sample size calculation was performed; the study was exploratory in design.

**Figure 1:**
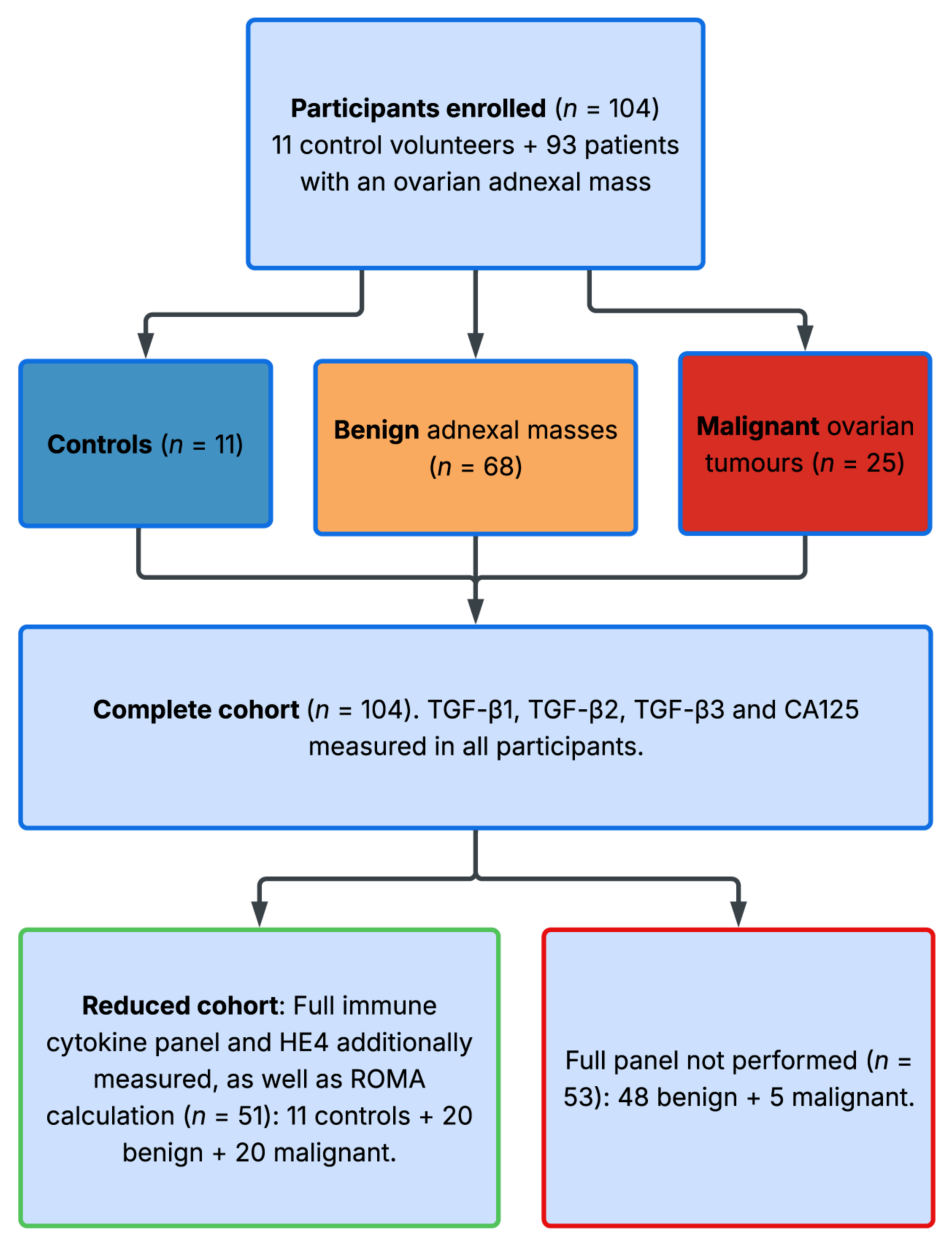
Participant flow diagram. A total of 104 participants were enrolled, comprising 11 control volunteers and 93 women with ovarian adnexal masses (68 benign, 25 malignant). TGF-β1, TGF-β2, TGF-β3, and CA125 were measured in all participants (complete cohort, *n* = 104). The full panel of 23 plasma cytokines and inflammatory molecules was additionally measured in a subset of 51 participants (reduced cohort: 11 controls, 20 benign, 20 malignant). Among participants with adnexal masses in this subset, HE4 and ROMA were also available. The remaining 53 participants were excluded for insufficient sample volume. All combined marker analyses, including the sequential algorithm and the linear predictor model, were performed exclusively within the reduced cohort.

Two analytic cohorts were defined based on available measurements (Figure 1). The complete cohort (*n* = 104; 11 controls, 68 benign, 25 malignant) comprised all participants with TGF-β isoform and CA125 measurements. The reduced cohort (*n* = 51; 11 controls, 20 benign, 20 malignant) comprised the subset of participants for whom the full panel of 23 plasma cytokines and inflammatory molecules was available; among participants with adnexal masses, HE4 measurement and ROMA calculation were also available. The remaining 53 participants were excluded from this subset due to insufficient sample volume. The clinicopathological characteristics of both cohorts are detailed in Table 1 and Supplementary Table S1, respectively. All other analyses, including the sequential algorithm and the linear predictor model, were performed exclusively within the reduced cohort to ensure analytical coherence across combined marker evaluations.

### 2.2. Acid Activation of Plasma for the Measurement of Total TGF-β1, TGF-β2, and TGF-β3

To quantify total TGF-β1, TGF-β2, and TGF-β3, plasma samples were acid-activated to dissociate the latent TGF-β complex. Briefly, 75 µL of 1 N HCl was added to 375 µL of plasma, and the samples were rocked for 10 min at 4 °C. The reaction was then neutralized with 75 µL of 1.2 N NaOH/0.5 M HEPES to halt the acid activation. The activated samples were stored on ice and assayed on the same day.

### 2.3. Enzyme-Linked Immunosorbent Assay (ELISA)

Plasma levels of TGF-β1 and TGF-β2 were measured using ELISA kits from R&D Systems (Minneapolis, MN, USA), and TGF-β3 was measured with a kit from Bioss Inc. (Woburn, MA, USA). Plasma concentrations of HE4 were measured using the Quantikine ELISA Human HE4/WFDC2 Immunoassay (R&D Systems). All assays were performed in duplicate according to the manufacturer’s instructions. For the TGF-β assays, acid-activated plasma samples were added to the microplate and incubated for 2 h at room temperature. Following a wash step, an enzyme-conjugated detection antibody was added and incubated for another 2 h, followed by a 30 min incubation with a chromogenic substrate. For all assays, reactions were stopped and colorimetrically quantified using a BioTek Synergy HT microplate reader (Cat. No. 7091000, BioTek Instruments Inc., Winooski, VT, USA). TGF-β plates were read at 540 nm, while HE4 plates were read at 450 nm with wavelength correction at 540 nm. Concentrations (expressed as pg/mL for cytokines and pmol/L for HE4) were calculated by comparing the optical density of the samples to an assay-specific standard curve generated during the assay

### 2.4. BioPlex Plasma Cytokine Measurement

Seven cytokines (IL-1α, IL-15, IL-1RA, IL-31, IL-7, TNF-β, and IFN-α) were measured in plasma using the ProcartaPlex™ Human Cytokine Panel 1C 7-Plex (Cat. No. EPX070-10010-901, Thermo Fisher Scientific, Waltham, MA, USA). The assay was performed according to the manufacturer’s instructions, including standards, controls, and plasma samples. Briefly, plasma was incubated with microbeads labeled with antibodies specific to each of the aforementioned cytokines for 120 min. After washing, the beads were incubated with a cocktail of detection antibodies, each specific to a single cytokine, for 60 min. Following an additional washing step, the beads were incubated with streptavidin-phycoerythrin for 15 min. Then, they were washed again and resuspended in an assay buffer for analysis using the Bio-Plex instrument (Bio-Rad, Hercules, CA, USA). The final concentration of interleukins was calculated using Bio-Plex Manager v5.0 software (Bio-Rad). All samples and standards were analyzed in triplicate. Plasma interleukin concentrations are expressed as pg/mL.

### 2.5. LEGENDplex Detection of Cytokines in Plasma

Plasma concentrations of IL-5, IL-13, IL-2, IL-6, IL-9, IL-10, IFN-γ, TNF-α, IL-17A, IL-17F, IL-4, and IL-22 cytokines were determined using the LEGENDplex^TM^ Human Th Cytokines Panel (12-plex) w/VbP V02 (Cat. No. 741028, BioLegend, San Diego, CA, USA). The capture beads from the kit were mixed and incubated with participant plasma samples according to the manufacturer’s instructions. The beads were differentiated by size and internal fluorescence intensity using a Cytek™ Aurora spectral flow cytometer (Cytek Biosciences, Fremont, CA, USA). The concentration of each analyte was determined from a standard curve generated within the same assay. Data were analyzed using the LEGENDplex™ Data Analysis Software Suite version 2025-05-01 which is available online (BioLegend; https://legendplex.qognit.com/user/login (accessed on 17 June 2026)). A total of 300 bead events were acquired for each type of cytokine, according to the manufacturer’s instructions. Results are expressed as pg/mL.

### 2.6. Statistical Analysis

All statistical analyses were performed using Python 3.13.3 with NumPy 2.3.4, pandas 2.2.3, matplotlib 3.10.3, seaborn 0.13.2, SciPy 1.16.0, and statsmodels 0.14.6. Intergroup differences in plasma molecule concentrations were evaluated using the Kruskal–Wallis test, followed by pairwise Dunn comparisons across all three diagnostic group pairs. Effect sizes were quantified as rank-based eta-squared (η^2^), interpreted according to Cohen’s conventional thresholds: small (η^2^ ≥ 0.01), medium (η^2^ ≥ 0.06), and large (η^2^ ≥ 0.14). Kruskal–Wallis and Dunn *p*-values were adjusted for multiple comparisons using the Benjamini–Hochberg (BH) false discovery rate (FDR) procedure; both raw *p*-values and adjusted q-values are reported throughout. Intergroup differences in clinicopathological characteristics were evaluated using the Kruskal–Wallis test for continuous variables, the chi-squared test or Fisher’s exact test for categorical variables, and the Mann–Whitney U test for variables available in two groups only; *p*-values for these comparisons were likewise adjusted using the Benjamini–Hochberg procedure. Statistical significance was set at α = 0.05 for all tests.

For each molecule and each binary comparison, ROC analysis was performed, and the AUC was calculated using the trapezoidal rule. The Youden index (J = sensitivity + specificity − 1) was applied to each ROC curve to identify the optimal concentration threshold, designated “t_low” for the Control vs. Benign boundary and “t_high” for the Benign vs. Malignant boundary. Bootstrap 95% confidence intervals for AUC were estimated using 1000 iterations with random resampling with replacement.

Based on the complementary pairwise patterns identified in the screening phase, TGF-β3 and IL-13 were selected for the development of two classification approaches within the reduced cohort. In the sequential algorithm, the Youden-optimized TGF-β3 threshold was applied at Step 1 to classify participants as TGF-β3-negative or TGF-β3-positive; TGF-β3-positive participants were routed to Step 2 where the Youden-optimized IL-13 threshold classified them as benign or malignant. Both thresholds were derived independently within the reduced cohort.

In the linear predictor model (LP), both markers were ln-transformed and entered as predictors into a proportional-odds ordinal logistic regression model fitted by maximum likelihood estimation via the L-BFGS-B algorithm, yielding a continuous linear predictor score defined as LP = β_1_ × ln(TGF-β3) + β_2_ × ln(IL-13). Two Youden-derived thresholds applied to the continuous LP score defined three diagnostic strata: LP < t_low (no lesion), t_low ≤ LP < t_high (benign), and LP ≥ t_high (malignant). Model coefficients are reported as β estimates with odds ratios, 95% confidence intervals, and *p*-values.

Apparent performance for the linear predictor was corrected for optimism using Harrell’s bootstrap optimism correction procedure [21]. One thousand bootstrap samples were drawn with replacement (*n* = 51) from the development cohort. In each replicate the proportional-odds model was refitted and both Youden-optimized thresholds (t_low, t_high) were re-derived de novo; optimism was estimated as the difference between performance in the bootstrap sample and performance when applied to the original cohort, averaged over all replicates, and subtracted from the apparent value. Correction was applied to the AUC, sensitivity, specificity and accuracy at the Youden thresholds, the three-class classification accuracy, and the sensitivity for malignancy. Bootstrap 95% confidence intervals for the AUC were obtained separately by the percentile method. The linear predictor was compared with CA125, HE4, and ROMA, using DeLong’s test on the apparent AUC values [22].

## 3. Results

### 3.1. Participant Characteristics and Study Flow

A total of 104 participants were enrolled, comprising 11 control volunteers without adnexal pathology and 93 women with ovarian adnexal masses (68 benign, 25 malignant). TGF-β isoforms and CA125 were measured in all 104 participants (complete cohort). The full molecule panel, HE4, and ROMA were available for a subset of 51 participants (reduced cohort: 11 controls, 20 benign, 20 malignant); the participant flow is shown in Figure 1. The clinicopathological characteristics of the complete and reduced cohorts are summarized in Table 1 and Supplementary Table S1, respectively. Women with malignant tumors were significantly older and more frequently postmenopausal than those with benign masses in the complete cohort (Table 1). Malignant tumors were predominantly of epithelial origin, with high-grade serous (24.0%), endometrioid (24.0%), clear cell (20.0%), and mucinous (20.0%) subtypes represented; 44.0% presented at FIGO stage I and 40.0% at stage III.

### 3.2. Screening of 23 Circulating Molecules Identifies 10 with Significant Intergroup Differences Across Diagnostic Categories

Plasma concentrations of 23 molecules were evaluated across three distinct diagnostic categories, controls without adnexal pathology, women with benign adnexal masses, and women with malignant ovarian tumors. The Kruskal–Wallis test with Dunn’s post hoc pairwise comparisons was used, with *p*-values adjusted by the Benjamini–Hochberg procedure; adjusted q-values are reported throughout.

**Figure 2:**
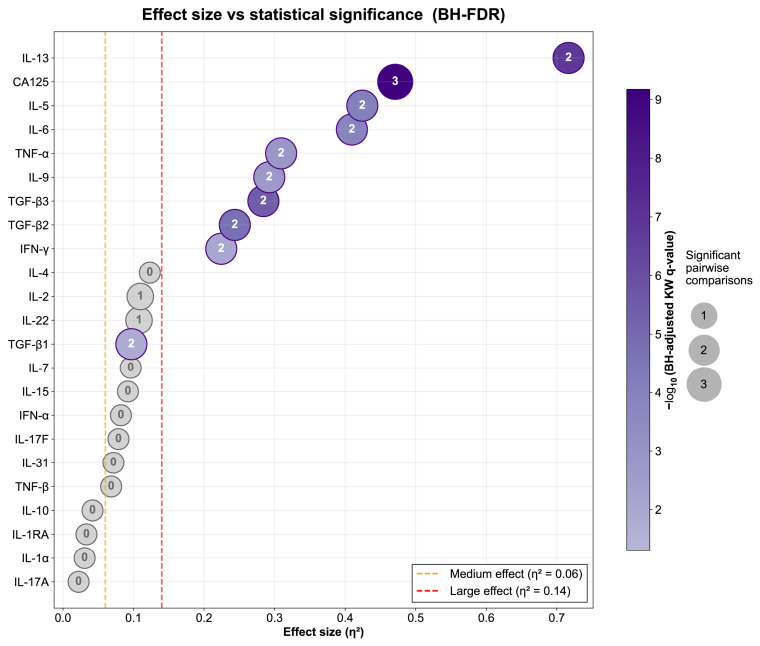
Statistical summary of circulating molecule screening across diagnostic categories. Bubble plot displaying the Kruskal–Wallis rank-based effect size (η^2^, x-axis) and statistical significance (−log_10_ BH-adjusted Kruskal–Wallis q-value, color gradient) for all 23 plasma molecules evaluated across controls, women with benign adnexal masses, and women with malignant ovarian tumors. Bubble size indicates the number of statistically significant pairwise BH-adjusted Dunn comparisons (0 to 3) among the three diagnostic groups. Colored bubbles denote molecules with Kruskal–Wallis q < 0.05 (*n* = 10); gray bubbles denote molecules with no significant Kruskal–Wallis q-value (*n* = 13). Dashed vertical lines indicate thresholds for medium (η^2^ = 0.06, orange) and large (η^2^ = 0.14, red) effect sizes according to Cohen’s conventions. TGF-β isoforms and CA125 were evaluated in the complete cohort (*n* = 104; 11 controls, 68 benign, 25 malignant); all remaining molecules were evaluated in the reduced cohort (*n* = 51; 11 controls, 20 benign, 20 malignant).

Figure 2 summarizes the Kruskal–Wallis effect size (η^2^) and BH-adjusted statistical significance of 23 molecules evaluated. Ten reached statistical significance (q < 0.05), with effect sizes ranging from η^2^ = 0.097 (TGF-β1) to η^2^ = 0.717 (IL-13); three additional molecules (IL-4, IL-2, and IL-22) reached nominal significance (*p* < 0.05) but did not survive correction for multiple comparisons (q = 0.073–0.079). The remaining 10 failed to reach omnibus significance and are provided in Supplementary Figure S1. The pairwise post hoc comparison patterns revealed functionally distinct behaviors that informed the organization of the following sections and are examined in detail below.

### 3.3. TGF-β Isoforms Identify Lesion Presence Without Malignancy Discrimination

The significant molecules were further characterized by examining their pairwise BH-corrected post hoc Dunn comparison profiles across the three diagnostic groups.

This analysis revealed that three molecules—TGF-β3, TGF-β2, and TGF-β1—showed significant concentration differences between controls and lesion-bearing women in both the Control vs. Benign and Control vs. Malignant comparisons, while the Benign vs. Malignant comparison remained non-significant for all three, identifying them as markers of lesion presence independent of malignant status.

To characterize their individual discriminatory capacity, ROC analysis was performed for each molecule across two clinically relevant binary comparisons: controls versus women with benign ovarian lesions (control vs. benign), and women with benign lesions versus women with malignant tumors (benign vs. malignant).

**Figure 3:**
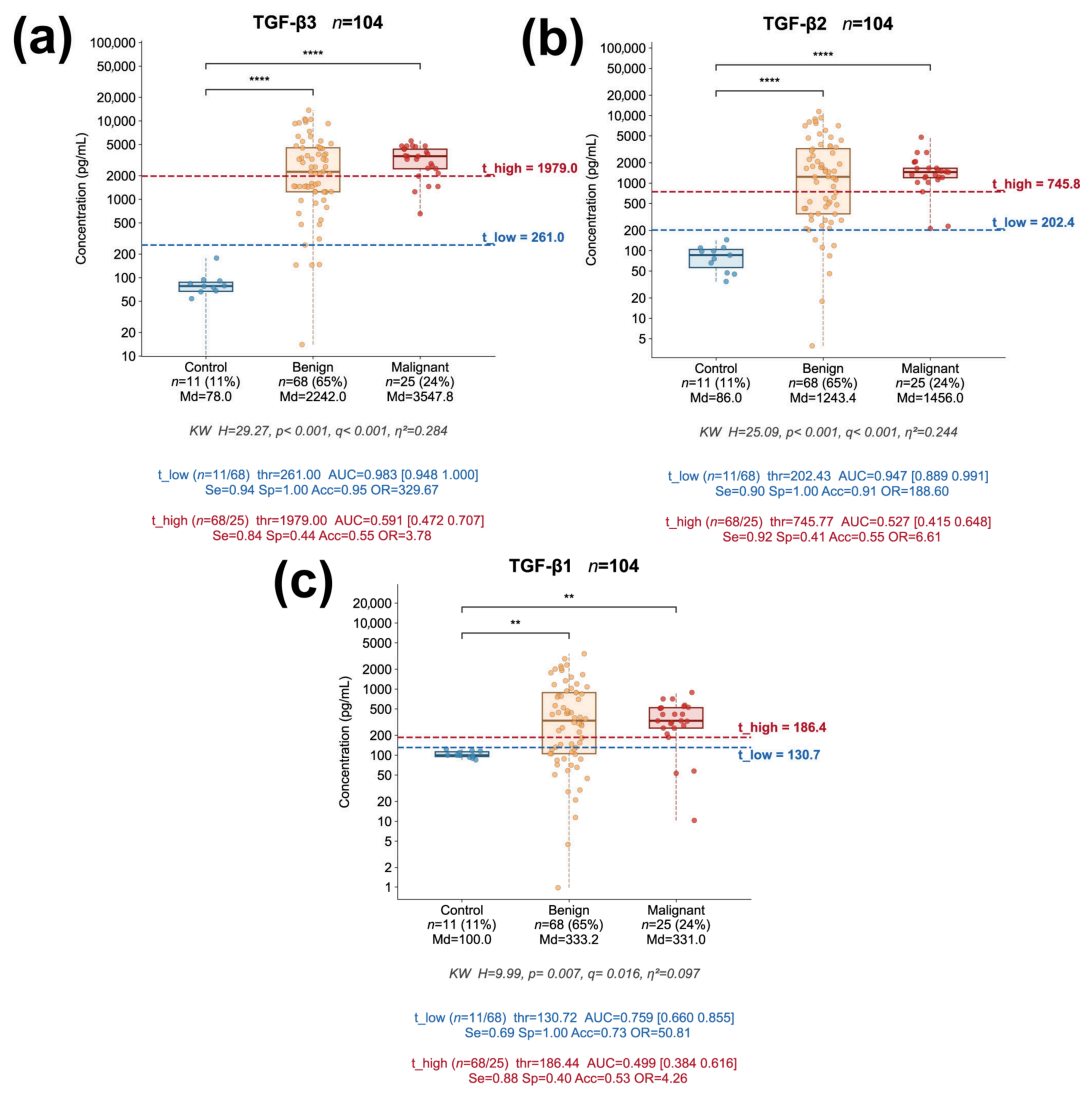
Circulating TGF-β isoform concentrations across diagnostic categories. Distribution plots for TGF-β3 (**a**), TGF-β2 (**b**), and TGF-β1 (**c**), evaluated in the complete cohort (*n* = 104; 11 controls, 68 benign, 25 malignant). Boxes represent the interquartile range with the median line; dashed whiskers extend to minimum and maximum values; individual data points are shown. Y-axes are displayed in logarithmic space. Blue dashed lines indicate the Youden-optimized threshold for the control vs. benign comparison (t_low); red dashed lines indicate the Youden-optimized threshold for the benign vs. malignant comparison (t_high). Significance brackets denote pairwise BH-adjusted Dunn q-values: ** q < 0.01, **** q < 0.0001. KW, Kruskal–Wallis H statistic; *p*, uncorrected Kruskal–Wallis *p*-value; q, BH-adjusted Kruskal–Wallis q-value; η^2^, rank-based effect size. Complete ROC-derived diagnostic metrics for each threshold are reported beneath each panel.

For each comparison, the Youden index was applied to identify optimal concentration thresholds for clinical diagnostic categories, designated as “t_low” at the control vs. benign boundary, and “t_high” at the benign vs. malignant boundary, shown as dashed lines in each panel. AUC with 95% bootstrap confidence intervals, sensitivity, specificity, and accuracy at each threshold are reported beneath each distribution, providing a holistic view of the discriminatory performance achieved at the two diagnostic boundaries. These three molecules are presented in Figure 3.

Among the TGF-β isoforms, TGF-β3 (Figure 3a) demonstrated the strongest discriminatory performance for lesion detection, with controls clustering below the lower threshold (t_low = 261.0 pg/mL), while both benign and malignant groups showed marked elevation, with substantial overlap between the two groups (Md = 2242.0 and 3547.8 pg/mL, respectively). Pairwise comparisons confirmed significant elevation in both lesion groups relative to controls (Control vs. Benign q < 0.0001; Control vs. Malignant q < 0.0001), yielding an AUC of 0.983 [0.948–1.000] for separating controls from women with ovarian lesions.

TGF-β2 (Figure 3b) followed the same pattern with significant separation from controls in both lesion groups (Control vs. Benign q < 0.0001; Control vs. Malignant q < 0.0001) and high lesion-detection capacity (AUC = 0.947 [0.889–0.991]).

TGF-β1 (Figure 3c) showed the weakest lower threshold performance in the isoform family (Control vs. Benign q = 0.006; Control vs. Malignant q = 0.006; AUC = 0.759 [0.660–0.855]; Se = 0.69), with greater distributional overlap between controls and the benign lesion groups and a sensitivity notably lower than its isoforms, suggesting a less pronounced biological response to lesion presence.

Across all three isoforms the benign vs. malignant comparison remained non-significant, with AUCs near chance (TGF-β3: 0.591; TGF-β2: 0.527; TGF-β1: 0.499), confirming that TGF-β isoform elevation reflects the presence of any ovarian lesion regardless of its malignant potential.

### 3.4. CA125 and TNF-α Show Progressive Concentration Gradients Across All Three Diagnostic Categories

**Figure 4:**
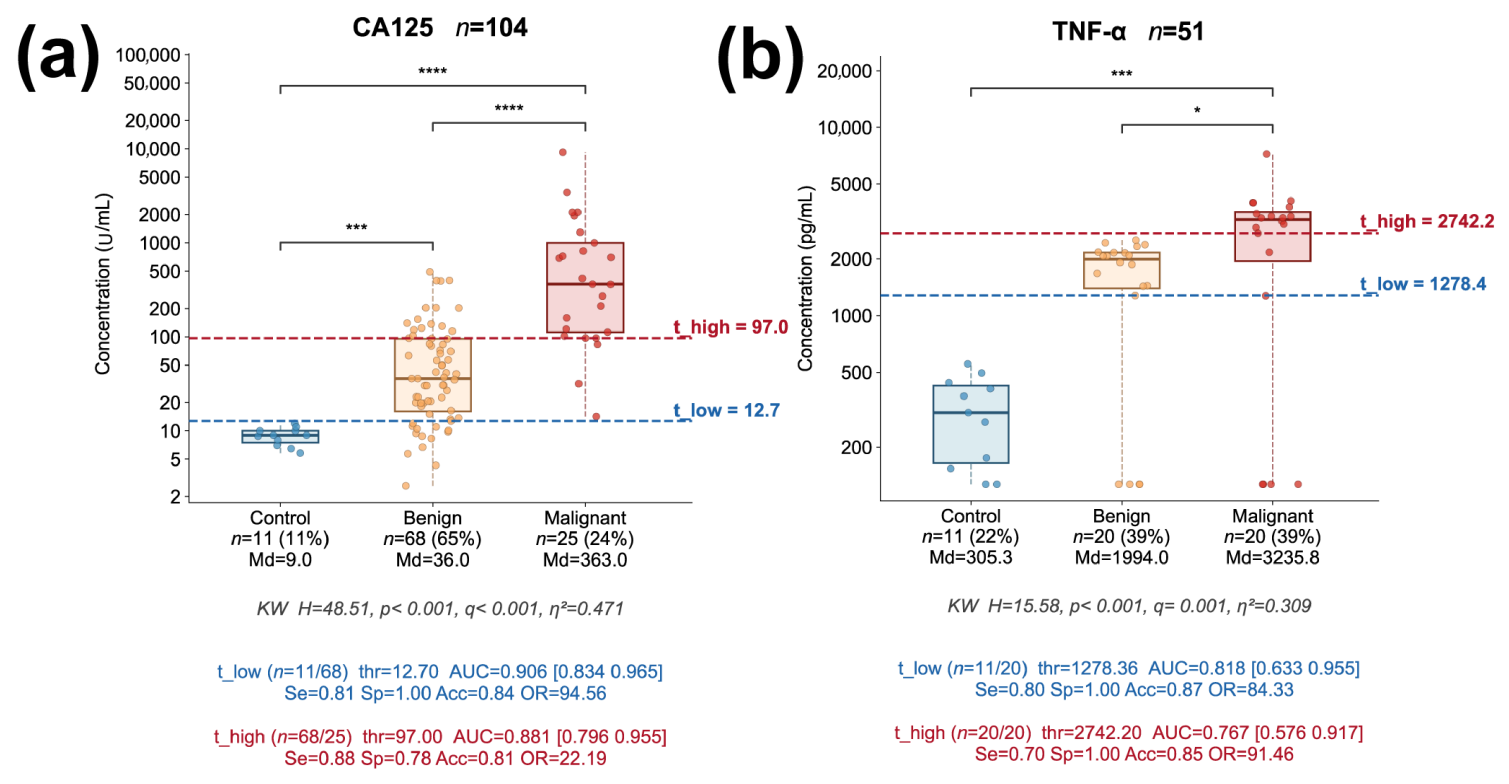
Circulating CA125 and TNF-α concentrations across diagnostic categories. Distribution plots for CA125 (**a**) and TNF-α (**b**). CA125 was evaluated in the complete cohort (*n* = 104; 11 controls, 68 benign, 25 malignant); TNF-α was evaluated in the reduced cohort (*n* = 51; 11 controls, 20 benign, 20 malignant). Significance brackets denote pairwise BH-adjusted Dunn q-values: * q < 0.05, *** q < 0.001, **** q < 0.0001. All plot conventions, threshold designations, and reported metrics are as described in Figure 3.

CA125 was the only molecule in the screening panel to reach statistical significance across all three pairwise comparisons (Control vs. Benign q < 0.001; Control vs. Malignant q < 0.001; Benign vs. Malignant q < 0.001), displaying a progressive concentration gradient from controls (Md = 9.0 U/mL) through benign (Md = 36.0 U/mL) to malignant tumors (Md = 363.0 U/mL) (Figure 4a). Its lesion-detection capacity was high (AUC = 0.906 [0.834–0.965]), and its capacity to separate malignant from benign cases was also meaningful (AUC = 0.881 [0.796–0.955]).

TNF-α (Figure 4b), evaluated in the reduced cohort, showed a similar progressive concentration gradient across the three diagnostic categories (Md = 305.3, 1994.0, and 3235.8 pg/mL for controls, benign, and malignant, respectively; KW H = 15.58, q < 0.001, η^2^ = 0.309). Pairwise comparisons confirmed significant elevation in the malignant group relative to both controls and benign cases (Control vs. Malignant q < 0.001; Benign vs. Malignant q = 0.034), while the Control vs. Benign comparison did not reach significance after correction (q = 0.050).

Despite their progressive concentration pattern, the distributions between benign and malignant groups remained meaningfully overlapping in both molecules, and neither achieved the malignancy-specific separation that would be required for confident preoperative tumor characterization.

### 3.5. IL-13, IL-5, IL-6, IL-9, and IFN-γ Are Specifically Elevated in Malignant Ovarian Tumors

**Figure 5:**
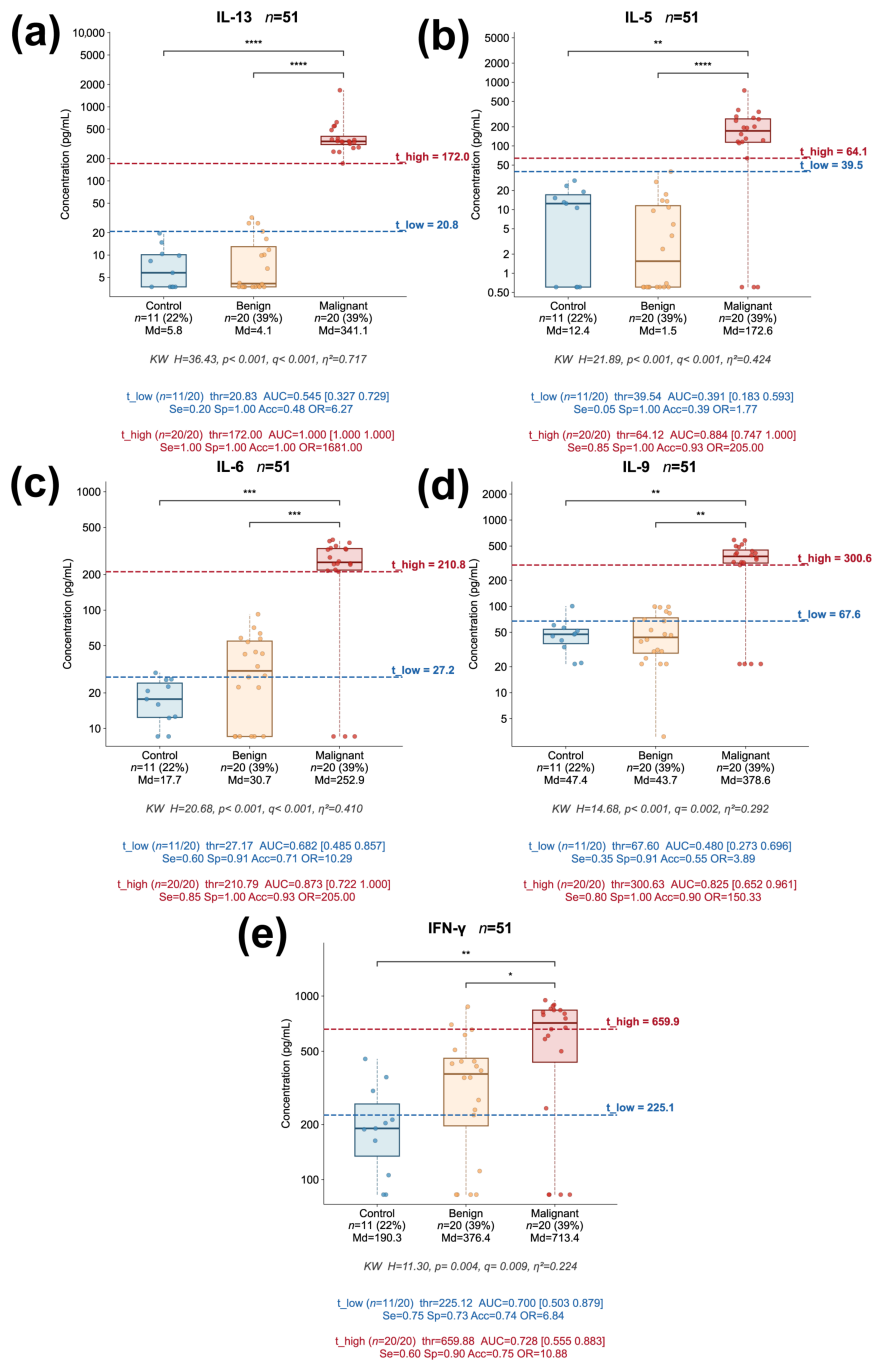
Plasma concentrations of malignancy-specific molecules across diagnostic categories. Distribution plots for IL-13 (**a**), IL-5 (**b**), IL-6 (**c**), IL-9 (**d**), and IFN-γ (**e**), all evaluated in the reduced cohort (*n* = 51; 11 controls, 20 benign, 20 malignant). Significance brackets denote pairwise BH-adjusted Dunn q-values: * q < 0.05, ** q < 0.01, *** q < 0.001, **** q < 0.0001. All plot conventions, threshold designations, and reported metrics are as described in Figure 3.

Five molecules evaluated in the reduced cohort—IL-13, IL-5, IL-6, IL-9, and IFN-γ—showed a pairwise pattern distinct from those previously reported. The Control vs. Benign comparison was non-significant in all five, while both the Control vs. Malignant and Benign vs. Malignant comparisons reached statistical significance, identifying them as molecules whose elevation is specific to malignant tumors rather than to a general ovarian lesion (Figure 5).

IL-13 (Figure 5a) showed the strongest and most specific malignancy signal in the entire screening panel, with controls (Md = 5.8 pg/mL) and benign (Md = 4.1 pg/mL) groups presenting nearly indistinguishable concentrations, while malignant tumors displayed a marked elevation (Md = 341.1 pg/mL), reflected in highly significant pairwise comparisons (Control vs. Malignant q < 0.001; Benign vs. Malignant q < 0.001; KW H = 36.43, q < 0.001, η^2^ = 0.717) and a perfect malignancy-separation capacity (AUC = 1.000) in this exploratory cohort.

IL-5 (Figure 5b) followed a similar pattern, with low concentrations in control (Md = 12.4 pg/mL) and benign (Md = 1.5 pg/mL) groups in contrast to a markedly elevated concentration in the malignant group with minimal overlap (Md = 172.6 pg/mL; Control vs. Malignant q = 0.003; Benign vs. Malignant q < 0.001; t_high AUC = 0.884 [0.747–1.000]).

IL-6 (Figure 5c) and IL-9 (Figure 5d) shared the same malignancy-specific pattern, with controls and benign groups showing no significant concentration differences, while malignant tumors were significantly elevated above non-malignant groups (IL-6: Control vs. Malignant q < 0.001, Benign vs. Malignant q < 0.001; IL-9: Control vs. Malignant q = 0.007, Benign vs. Malignant q = 0.001) yielding strong malignancy-separation capacities (IL-6: t_high AUC = 0.873 [0.722–1.000]; IL-9: t_high AUC = 0.825 [0.652–0.961]).

IFN-γ (Figure 5e) showed the most modest signal within this group, with a significant elevation in concentration in malignant tumors relative to control and benign groups (Control vs. Malignant q = 0.004; Benign vs. Malignant q = 0.043), and moderate malignancy-separation capacity (t_high AUC = 0.728 [0.555–0.882]).

### 3.6. IL-4, IL-2, and IL-22 Show Nominally Malignancy-Associated Elevation

**Figure 6:**
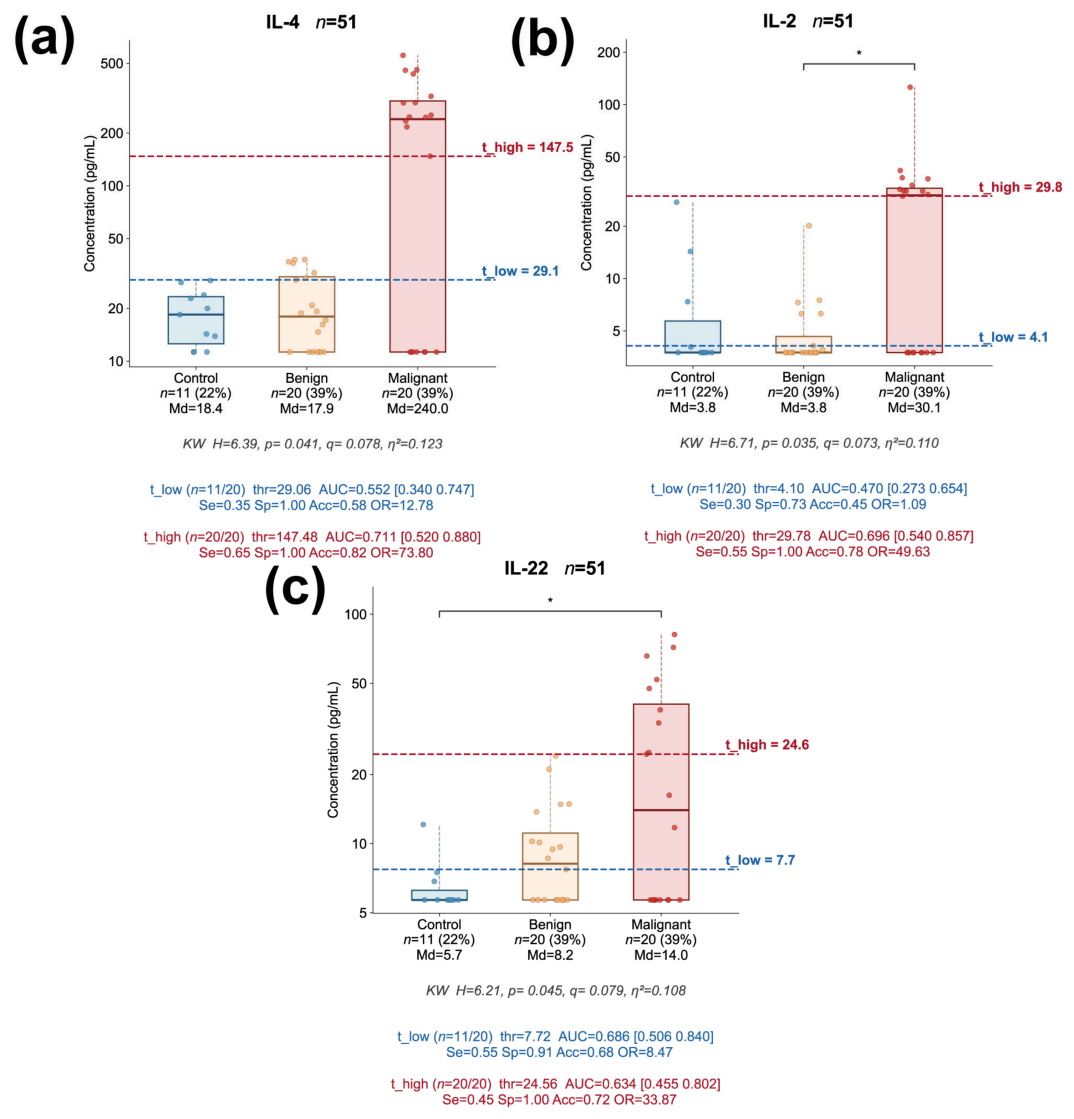
Circulating IL-4, IL-2, and IL-22 concentrations across diagnostic categories. Distribution plots for IL-4 (**a**), IL-2 (**b**), and IL-22 (**c**), all evaluated in the reduced cohort (*n* = 51; 11 controls, 20 benign, 20 malignant). Significance brackets denote pairwise BH-adjusted Dunn q-values: * q < 0.05. All plot conventions, threshold designations, and reported metrics are as described in Figure 3.

Three additional molecules reached nominal Kruskal–Wallis significance (*p* < 0.05) but did not survive correction for multiple comparisons (IL-4 q = 0.078; IL-2 q = 0.073; IL-22 q = 0.079). All three showed higher median concentrations in the malignant group relative to controls and benign cases (Figure 6). IL-2 showed a significant pairwise elevation in malignant tumors relative to benign cases (Dunn q = 0.046; t_high AUC = 0.696 [0.540–0.857]), while IL-22 showed significant elevation in malignant tumors relative to controls (Dunn q = 0.040; t_high AUC = 0.634 [0.455–0.802]). IL-4 did not reach significance in any individual pairwise comparison after correction (t_high AUC = 0.711 [0.520–0.880]). Given the modest effect sizes (η^2^ = 0.108–0.123) and the failure to survive omnibus correction, these observations should only be regarded as hypothesis-generating.

### 3.7. A Sequential TGF-β3/IL-13 Algorithm Achieves 96.1% End-to-End Accuracy in the Reduced Cohort

**Figure 7:**
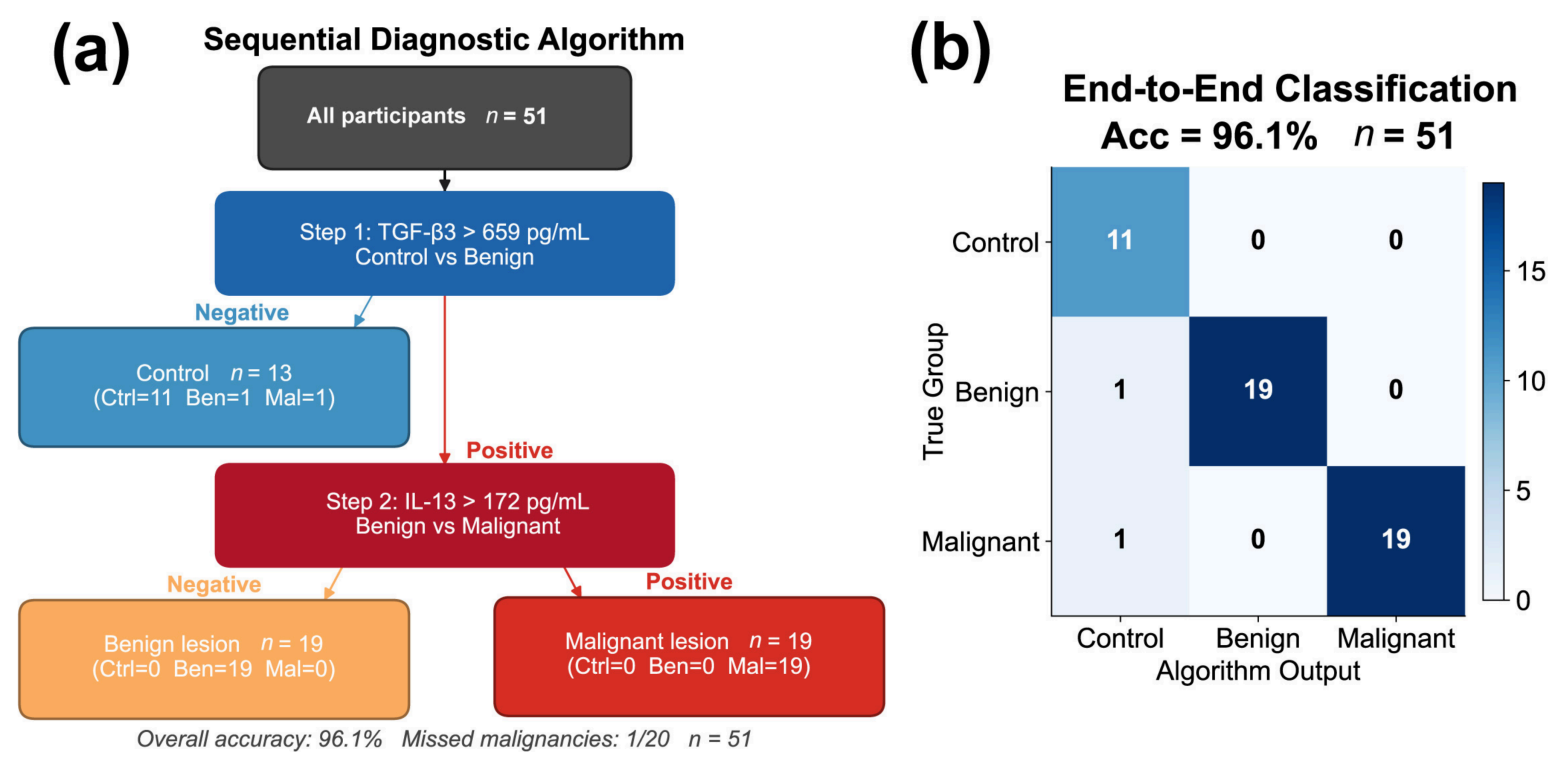
Sequential TGF-β3/IL-13 diagnostic algorithm and end-to-end classification performance. (**a**) shows the algorithm flowchart with participant counts at each decision node. Step 1 applies a TGF-β3 threshold of 659.0 pg/mL to separate participants with no ovarian lesion from those with an ovarian lesion; TGF-β3-positive individuals are routed to Step 2, which applies an IL-13 threshold of 172.0 pg/mL to distinguish benign cases from malignant tumors. *n* = indicates the number of participants routed to each terminal category; numbers in parentheses indicate the true diagnostic composition of each node. (**b**) shows the end-to-end confusion matrix for all 51 individuals in the reduced cohort. Overall accuracy and cohort size are indicated in the figure title.

Findings from Section 3.3 and Section 3.5 identified TGF-β3 and IL-13 as functionally complementary markers, where TGF-β3 concentration can detect the presence of any ovarian lesion without discriminating between benign and malignant disease, and IL-13 is specifically elevated in malignant tumors relative to controls and benign cases. This pattern motivated the development of a sequential two-step diagnostic algorithm in which TGF-β3 screens for lesion presence at Step 1, and IL-13 confirms malignancy at Step 2 among TGF-β3-positive participants. Both thresholds were derived independently within the reduced cohort (*n* = 51) using the Youden index, which accounts for the shift in the TGF-β3 optimal threshold observed in the complete cohort. The algorithm and its end-to-end classification performance are presented in Figure 7.

At Step 1, TGF-β3 screening routed 38 of 51 participants to Step 2 as TGF-β3-positive (TGF-β3 > 659 pg/mL), while 13 were classified as no lesion, 11 of whom were true controls, with one benign and one malignant case falling below the Step 1 threshold, as shown in the flowchart (Figure 7a). Among the 38 individuals routed to Step 2, IL-13 concentrations above 172.0 pg/mL correctly classified all evaluated cases, 19 as malignant and 19 as benign, with no misclassifications at this stage. However, as shown in the end-to-end confusion matrix (Figure 7b), one malignant case had been gated out at Step 1 and never reached IL-13 evaluation, resulting in an overall accuracy of 96.1% across all 51 participants and a missed malignancy rate of 1 in 20. Both errors in the algorithm originate exclusively at Step 1, reflecting the inherent limitation of sequential gating: individuals misclassified at the screening step are excluded from the confirmatory evaluation regardless of their true diagnostic status.

### 3.8. A TGF-β3/IL-13 Linear Predictor Score Achieves 98.0% Classification Accuracy with Zero Missed Malignancies

Although the sequential algorithm demonstrated that TGF-β3 and IL-13 together classify ovarian adnexal masses with high accuracy, its stepwise design carries an inherent limitation: individuals misclassified at Step 1 are excluded from subsequent evaluation regardless of their true diagnostic status.

To address this, both markers were ln-transformed and entered simultaneously as predictors into a proportional-odds ordinal logistic regression model within the reduced cohort. In this model, each predictor is assigned a coefficient (β) estimated by maximum likelihood, and the linear predictor score is defined as their weighted sum: LP = β1 × ln(TGF-β3) + β2 × ln(IL-13). Both coefficients were statistically significant and positively associated with higher diagnostic category, yielding LP = 3.427 × ln(TGF-β3) + 3.254 × ln(IL-13). Higher plasma concentrations of either marker are independently associated with higher diagnostic category in this ordinal model, reflecting their complementary contributions to the separation of the three groups observed in the LP score distribution. Complete coefficient estimates with 95% confidence intervals are provided in Table 2.

**Figure 8:**
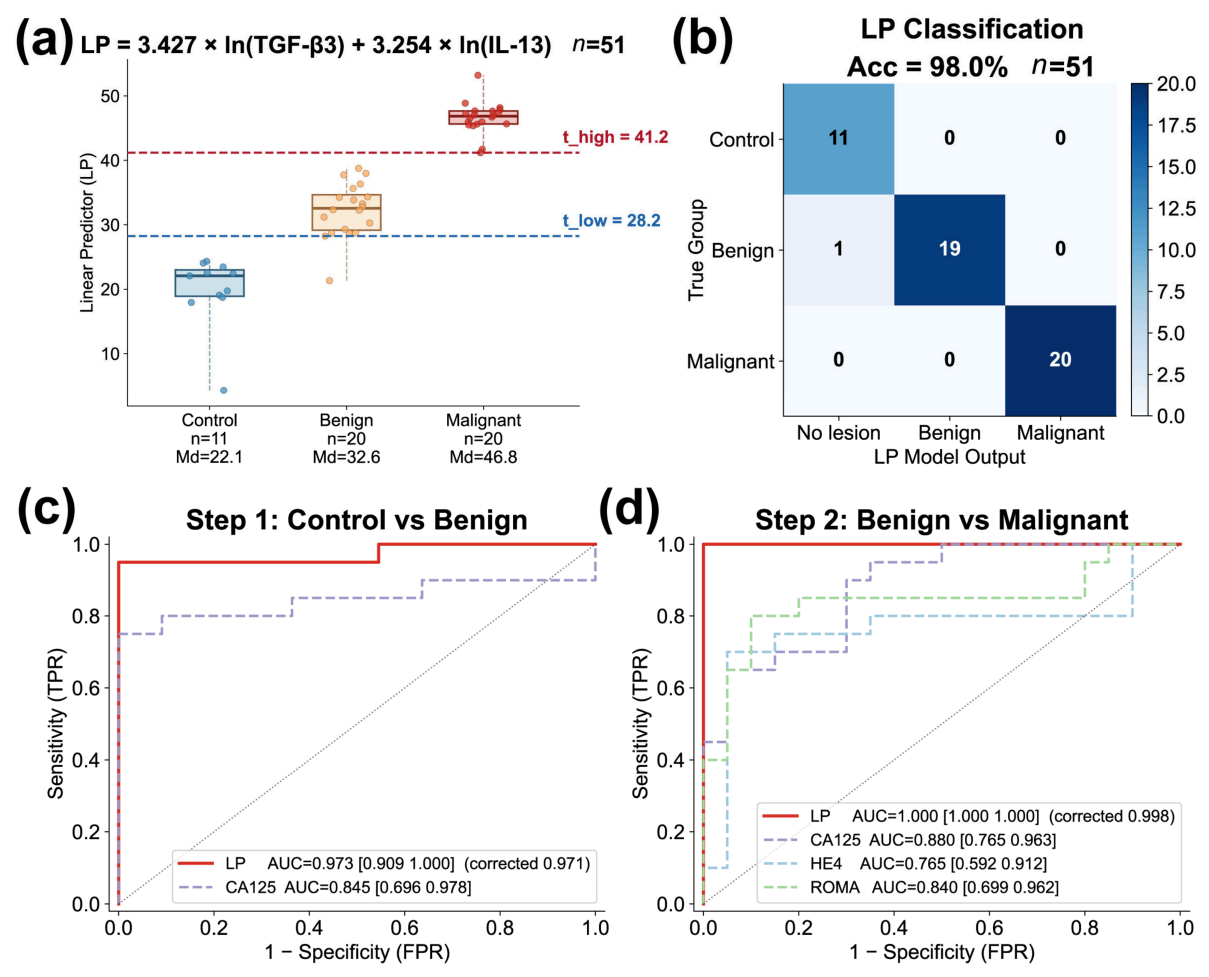
TGF-β3/IL-13 linear predictor score distribution, ROC performance, and classification accuracy. (**a**) Distribution of the linear predictor score across the three diagnostic groups in the reduced cohort. Boxes represent the interquartile range with the median line; individual data points for each subject are shown. Blue and red dashed lines indicate the Youden-optimized thresholds t_low and t_high, respectively. (**b**) Apparent LP classification confusion matrix for all 51 individuals. (**c**,**d**) show ROC curves for the Control vs. Benign and Benign vs. Malignant comparisons, respectively. In both panels, the LP score is displayed as a solid red line with apparent and optimism-corrected AUCs, and CA125 is included as a baseline comparator (purple dashed line). (**d**) additionally displays HE4 and ROMA as comparators (blue and green dashed lines, respectively), each with AUC and 95% bootstrap confidence intervals. The diagonal gray line represents random-level discrimination (AUC = 0.500).

The LP score produced three visually non-overlapping distributions across the three diagnostic groups (Figure 8a), with median LP values of 22.1, 32.6, and 46.8 for controls, benign, and malignant respectively. Two Youden-derived thresholds applied to the continuous LP score defined three diagnostic strata: participants below t_low = 28.2 classified as no lesion, participants between t_low and t_high = 41.2 classified as benign, and participants above t_high classified as malignant. Controls clustered below t_low, malignant cases clustered above t_high, and benign cases occupied the intermediate zone with only one exception, a single benign case falling below t_low, resulting in an apparent overall classification accuracy of 98.0%, with zero missed malignancies (Figure 8b).

ROC analysis of the LP score across the two binary comparisons demonstrated high discriminatory capacity at both diagnostic boundaries (Figure 8c,d). For the Control vs. Benign comparison, the LP achieved an apparent AUC of 0.973 [0.909–1.000] (Bootstrap-corrected 0.971), nominally outperforming CA125 at the same boundary (AUC = 0.845 [0.696–0.978]). For the Benign vs. Malignant comparison, the LP achieved near-perfect separation in this exploratory cohort (AUC = 1.000 [1.000–1.000], corrected 0.998), significantly outperforming CA125 (AUC = 0.880 [0.765–0.963]; DeLong *p* = 0.033), HE4 (AUC = 0.765 [0.592–0.912]; DeLong *p* = 0.002), and ROMA (AUC = 0.840 [0.699–0.962]; DeLong *p* = 0.013). Complete apparent and optimism-corrected diagnostic metrics and DeLong comparisons are provided in Table 3.

## 4. Discussion

The preoperative discrimination of benign from malignant ovarian adnexal masses remains one of the central diagnostic challenges in gynecological oncology, as current standard tools rely on a limited number of tumor-associated markers that are often unable to capture the complexity of ovarian pathology. On the biological side, the tumor microenvironment of ovarian cancer is characterized by a systemic immune dysregulation involving the reciprocal suppression of Th1-mediated cytotoxic responses and the amplification of Th2-associated immunosuppressive signaling, a shift that extends beyond the peritoneal cavity and is detectable in the peripheral circulation [10]. Thus, we hypothesized that a broad profiling approach of circulating plasma molecules encompassing distinct biological groups, including TGF-β isoforms, Th1 effector cytokines, Th2/M2-associated mediators, and pleiotropic inflammatory molecules, could identify candidate markers whose circulating concentrations reflect the immunological state associated with lesion presence, malignant transformation, or both and that could complement existing tools for preoperative risk stratification.

Among the TGF-β family members, all three isoforms showed significant elevation in both lesion groups relative to controls, while remaining non-significantly different between the two lesion types. At the cellular level, this shared pattern is consistent with their functional equivalence: all three isoforms promote ovarian cancer cell migration and invasion through a Smad3-dependent mechanism involving MMP secretion and E-cadherin downregulation [14], while independently driving cell migration via ZEB1 upregulation and N-cadherin induction without affecting proliferation [23]. However, this functional equivalence at the receptor level contrasts with both their differential tissue prevalence—TGF-β2 and TGF-β3 are overexpressed in 66% of malignant ovarian tumors compared with 44% for TGF-β1 [14], and the differential magnitude of their circulating signals, with TGF-β3 showing the strongest lesion-detection capacity (t_low AUC = 0.983), followed by TGF-β2 (t_low AUC = 0.947) and TGF-β1 (t_low AUC = 0.759). A potential isoform-specific explanation for this hierarchy emerges from the work of Wu et al., who demonstrated that COL11A1 overexpression in ovarian cancer cells activates cancer-associated fibroblasts specifically through TGF-β3 via the NF-κB/IGFBP2 axis, without altering TGF-β1 or TGF-β2 expression or promoter activity; a TGF-β3 antibody abrogated CAF activation and prevented tumor formation in vivo, and high TGF-β3 tissue levels were associated with poor patient survival [24]. Since CAF activation is a feature of the ovarian stromal microenvironment that may precede malignant transformation [25], this TGF-β3-specific production mechanism offers a biologically coherent explanation for why TGF-β3 produces the strongest circulating signal across both benign and malignant lesion groups. Consistent with these isoform-specific roles, Zhou et al. reported that elevated expression of TGF-β2 and TGF-β3 mRNA was associated with unfavorable prognosis in serous, poorly differentiated, and late-stage ovarian carcinoma [15], while TGF-β1 showed no prognostic association. Collectively, these findings suggest that isoform-specific TGF-β3 quantification in circulation may capture biologically meaningful information that aggregate measurements do not.

Five molecules in our screening panel—IL-13, IL-5, IL-6, IL-9, and IFN-γ—shared a consistent pattern of significant elevation in malignant tumors relative to both controls and benign cases, with no clear distinction between the two non-malignant groups, identifying them as malignancy-specific markers. Among these, IL-13 showed the most striking signal in the entire screening panel, with malignant tumors displaying complete separation from control and benign cases at the upper threshold in our exploratory cohort (AUC = 1.000). The absence of IL-13 elevation in benign cases is particularly notable; it suggests that the IL-13 signal is not a general response to an ovarian lesion but is specifically activated by malignant transformation. This observation is consistent with a receptor-gated mechanism: IL-13Rα2 is expressed in 83% of epithelial ovarian cancer specimens but is absent or barely detectable in normal ovarian tissue [26], with no significant correlation between IL-13Rα2 expression and FIGO stage, indicating that the receptor is present across the disease spectrum rather than restricted to advanced cases [26]. When this receptor is present, IL-13 promotes ovarian cancer invasion and metastasis through IL-13Rα2-mediated ERK/AP-1 signaling and MMP induction; critically, this pathway is activated exclusively in IL-13Rα2-positive cells, and even endogenous IL-13 is sufficient to drive metastasis in IL-13Rα2-positive tumors in vivo [27]. At the tissue level, Ripley et al. demonstrated that IL-13 mRNA and protein are expressed in ovarian carcinoma at higher levels than in normal ovarian tissue, with expression increasing with disease stage and localized in both tumor cells and infiltrating inflammatory cells [28], consistent with a dual-source, malignancy-associated IL-13 signal whose circulating reflection is gated by the presence of the IL-13Rα2 signaling axis in the transformed epithelium.

The remaining significant molecules displayed pairwise patterns that, while not incorporated into the final model, informed the marker selection process and warrant acknowledgment as hypothesis-generating observations. CA125 was the only molecule to reach significance across all three pairwise comparisons, consistent with its established role as a broad ovarian tumor marker whose elevation reflects a range of pathological states. TNF-α displayed a similar progressive concentration gradient across the three diagnostic categories, though its lower boundary comparison did not reach significance after correction (Dunn q = 0.050); its malignancy-associated elevation is consistent with prior reports of elevated serum TNF-α in ovarian cancer [29], and with the evidence that the proportion of TNF-α-positive samples is higher in ovarian cancer than in benign ovarian disease at the tissue level [20]. IL-5, IL-6, and IL-9 followed the same malignancy-specific pattern as IL-13, with progressively attenuated discriminative performance, consistent with documented elevation of serum IL-5 and IL-6 in ovarian cancer [16], the independent association of IL-6 with tumor burden and reduced survival across multiple cohorts [30], and the upregulation of serum IL-9 (AUC = 0.870) in an independent ovarian cancer cohort [17]. IFN-γ showed the most modest malignancy-specific signal, consistent with its paradoxical pro-tumorigenic role via HLA-E upregulation in the ovarian tumor microenvironment [18] and its identification as part of a cytokine signature associated with chemotherapy resistance [17]. Its malignancy-associated elevation at the benign vs. malignant boundary in our cohort is thus consistent with the pro-tumorigenic activity documented at the tissue level, suggesting that the concurrent activation of Th1 signaling alongside the dominant Th2/M2 shift may be a systemic feature of ovarian malignancy that warrants further characterization in larger independent cohorts. Three additional molecules, IL-4, IL-2, and IL-22, reached nominal Kruskal–Wallis significance (*p* < 0.05) but did not survive Benjamini–Hochberg correction (q = 0.073–0.079); their malignancy-associated elevation patterns are consistent with prior reports but require confirmation in larger cohorts [31,32,33,34,35].

The development of multiparametric serum models for preoperative discrimination of adnexal masses has been an active area of research, with logistic regression-based approaches combining markers such as CA19-9, CA125, NLR, PLR, and BDNF [36], or inflammatory ratios alongside CA125 [37] consistently demonstrating improved discrimination over individual markers alone. Moore et al. demonstrated that while combining eight serum markers (CA125, HE4, YKL-40, transthyretin, ApoA1, Beta-2-microglobulin, transferrin, and LPA) achieved an AUC of 0.944 for epithelial ovarian cancer detection, the gain over CA125/HE4 combination alone (AUC = 0.912) was minimal and did not justify clinical adoption [38], supporting the notion that biological complementarity between a small number of markers may be a more efficient strategy than panel expansion. A previous study conducted at our institution demonstrated that multiparametric classifiers integrating circulating serum molecules including MRP8/14, OPN, SAA, IL-6, IL-8 and IGFBP-4 alongside established tumor markers improved diagnostic performance for ovarian adnexal mass characterization beyond what individual markers could achieve [39]. Building on this evidence, we took a screening approach from which TGF-β3 and IL-13 emerged as biologically complementary candidate markers, addressing distinct diagnostic boundaries, TGF-β3 discriminating lesion-bearing women from controls regardless of malignant status, and IL-13 specifically elevated in malignant tumors relative to both non-malignant groups. Of these two contributions, the malignancy-discrimination task, represents the primary clinically relevant preoperative question, and is addressed by the IL-13 component and the combined LP model. Their sequential combination into a two-step diagnostic algorithm achieved 96.1% end-to-end accuracy in the reduced cohort. The proportional-odds ordinal logistic regression model, which addressed the inherent limitation of sequential gating by combining both markers simultaneously into a continuous LP score, achieved an apparent 98.0% accuracy with zero missed malignancies. Harrell’s bootstrap optimism correction confirmed that these results are not substantially inflated by in-sample fitting, yielding corrected values of 97.2% malignancy sensitivity, and a corrected AUC of 0.998 at the benign vs. malignant boundary. In head-to-head comparison with all four measurements available, the LP significantly outperformed CA125 (DeLong *p* = 0.033), HE4 (*p* = 0.002), and ROMA (*p* = 0.013) at the malignancy-separation boundary, while the advantage over CA125 at the lesion-detection boundary did not reach significance (DeLong *p* = 0.089). Notably, the LP’s superiority at the malignancy boundary persists whether the apparent (AUC = 1.000) or optimism-corrected (AUC = 0.998) value is considered, as all three comparator AUCs fall well below the corrected estimate.

Several limitations of this study must be acknowledged. First, no formal sample size calculation was performed; the study was exploratory in design. Benign and malignant samples were collected at a single tertiary referral hospital from participants already scheduled for surgical intervention, where the proportion of malignant cases is higher than in general preoperative settings. The control group was limited to 11 control volunteers without adnexal pathology, which renders specificity estimates at the lesion detection boundary especially sensitive to small sample fluctuations. The wide confidence intervals on the model’s odds ratios similarly reflect the limited sample size and should be interpreted with caution, even though the direction and significance of both coefficients were consistent. Marker selection and model development were performed in the same reduced cohort; however, Harrell’s bootstrap optimism correction confirmed minimal performance inflation, and the Benjamini–Hochberg procedure was applied to the omnibus screening to control for multiple comparisons. Furthermore, the 53 participants excluded from the reduced cohort due to insufficient sample volume comprised 48 benign and 5 malignant cases, resulting in a higher proportion of malignant cases in the reduced cohort than in the complete cohort; this enrichment may have influenced model development and performance estimation and should be considered when interpreting the reported metrics. Nonetheless, all reported metrics should be interpreted as internally validated exploratory performance. The head-to-head comparison with CA125, HE4, and ROMA provides initial evidence of the LP’s competitive performance at the malignancy-separation boundary, but the model was not compared against composite clinical risk stratification tools such as RMI, ADNEX, or the IOTA simple rules; the proposed model should therefore be regarded as a candidate approach whose incremental diagnostic value over these established tools requires formal prospective evaluation. Even so, the biological complementarity of TGF-β3 and IL-13, one reflecting lesion presence across the full diagnostic spectrum and the other specifically elevated in malignant disease, provides a mechanistically grounded rationale for their combined use that extends beyond the performance observed in this single cohort. Prospective validation in larger independent cohorts with particular attention to clinical subgroups where CA125 performs poorly, including premenopausal women and early-stage disease, is needed to establish whether this two-marker approach offers additive diagnostic value in a real-world preoperative setting.

## 5. Conclusions

The present study provides evidence supporting the hypothesis that systematic profiling of circulating plasma molecules in women with ovarian adnexal masses can identify biologically motivated diagnostic candidates. TGF-β3 and IL-13 showed complementary behavior, reflecting lesion presence and malignancy, respectively. Their combination in an ordinal regression model showed minimal optimism upon bootstrap correction and significantly outperformed CA125, HE4, and ROMA at the malignancy-separation boundary in our exploratory cohort. Together, these findings represent a proof-of-concept whose prospective evaluation in larger, multicenter independent cohorts is warranted to establish whether this two-marker approach offers additive value for preoperative risk stratification of ovarian adnexal masses.

## Figures and Tables

**Table 1:** Clinicopathological characteristics of the complete cohort.

Characteristic	Control	Benign	Malignant	Test	*p* (Raw)	q (BH-adj)
*n*	11	68	25			
Age, years. Median [IQR]	47.0 [43.0–51.5]	40.0 [33.0–51.2]	51.0 [44.0–56.0]	KW	0.002	0.002
Age at menarche, years. Median [IQR]	12.0 [11.0–12.5]	13.0 [11.0–13.0]	12.0 [11.0–13.2]	KW	0.409	0.443
Parity. Median [IQR]	2.0 [1.0–2.0]	1.0 [0.0–3.0]	1.5 [1.0–3.0]	KW	0.870	0.870
Nulliparous, *n* (%)	0 (0.0%)	20 (29.4%)	4 (16.0%)	χ^2^	0.063	0.081
Postmenopausal, *n* (%)	8 (72.7%)	16 (23.5%)	15 (60.0%)	χ^2^	<0.001	<0.001
Age at menopause, years. Median [IQR]	42.0 [41.0–42.2]	50.0 [48.0–51.2]	48.0 [42.0–49.8]	KW	<0.001	<0.001
CA125, U/mL. Median [IQR]	9.0 [7.5–10.0]	36.0 [16.1–95.3]	363.0 [112.0–1000.0]	KW	<0.001	<0.001
HE4, pmol/L. Median [IQR]	—	109.5 [81.0–140.9]	198.8 [151.5–226.0]	MW	<0.001	<0.001
ROMA, %. Median [IQR]	—	28.5 [20.1–43.1]	73.7 [60.5–88.2]	MW	<0.001	<0.001
Largest tumor diameter, cm. Median [IQR]	—	7.1 [5.9–9.9]	11.7 [7.2–16.9]	MW	0.001	0.002
Bilateral, *n* (%)	—	11 (16.2%)	6 (24.0%)	Fisher	0.381	0.443
Histological subtype, *n* (%)						
Endometrioma/endometriosis	—	28 (41.2%)	—			
Serous cystadenoma	—	13 (19.1%)	—			
Mucinous cystadenoma	—	4 (5.9%)	—			
Mature teratoma	—	11 (16.2%)	—			
Other benign	—	12 (17.6%)	—			
High-grade serous	—	—	6 (24.0%)			
Low-grade serous	—	—	1 (4.0%)			
Endometrioid	—	—	6 (24.0%)			
Clear cell	—	—	5 (20.0%)			
Mucinous	—	—	5 (20.0%)			
Other malignant	—	—	2 (8.0%)			
FIGO stage (malignant), *n* (%)						
Stage I	—	—	11 (44.0%)			
Stage II	—	—	2 (8.0%)			
Stage III	—	—	10 (40.0%)			
Stage IV	—	—	1 (4.0%)			
Unspecified	—	—	1 (4.0%)			
Tumor grade (malignant), *n* (%)						
High grade	—	—	11 (44.0%)			
Low grade	—	—	1 (4.0%)			
Grade not specified	—	—	13 (52.0%)			

Continuous variables are reported as median [interquartile range, IQR]; categorical variables as *n* (%). Intergroup comparisons were performed using the Kruskal–Wallis test (KW) for continuous variables, the chi-squared test (χ^2^) or Fisher’s exact test for categorical variables, and the Mann–Whitney U test (MW) for variables available in two groups only. *p* (raw), unadjusted *p*-value; q (BH-adj), Benjamini–Hochberg adjusted q-value; FIGO, International Federation of Gynecology and Obstetrics; CA125, cancer antigen 125; HE4, human epididymis protein 4; ROMA, Risk of Ovarian Malignancy Algorithm. HE4 and ROMA were available only in the reduced cohort and are therefore reported for benign and malignant groups only. Dashes (—) indicate variables not applicable in that particular group. Women with adnexal masses were enrolled on the basis of a preoperatively identified adnexal mass on transvaginal ultrasonography and confirmed histologically at surgery. Control volunteers had no adnexal pathology confirmed by transvaginal ultrasonography and CA125 measurement. HE4 and ROMA were available for participants with adnexal masses in the reduced cohort only and are therefore not reported for control volunteers. Other benign (*n* = 12) comprises minor epithelial-lined cysts (paratubal, simple, inclusion, and atrophic cysts; *n* = 7), non-epithelial and mesenchymal lesions (leiomyoma, fibroma, and ovarian stroma with cystic degeneration; *n* = 4), and one inflammatory lesion (granulomatous reaction secondary to pelvic inflammatory disease; *n* = 1). Other malignant (*n* = 2) comprises one adenocarcinoma not otherwise specified (epithelial origin) and one adult granulosa cell tumor (sex-cord/stromal origin).

**Table 2:** Ordinal logistic regression model coefficients.

Parameter	β	OR	CI Low	CI High	z	*p*
ln(TGF-β3)	3.427	30.790	1.989	476.646	2.452	0.014
ln(IL13)	3.254	25.900	2.423	276.903	2.692	0.007

β, estimated regression coefficient; OR, odds ratio; CI low and CI high, lower and upper bounds of the 95% confidence interval of the odds ratio; z, Wald z-statistic; *p*, two-sided *p*-value. Both coefficients represent the change in log-odds of belonging to a higher diagnostic category per unit increase in the natural logarithm of the respective marker concentration.

**Table 3:** Diagnostic performance of the TGF-β3/IL-13 linear predictor score and comparator markers in the reduced cohort.

Comparison	Ctrl vs. Ben	Ctrl vs. Ben	Ben vs. Mal	Ben vs. Mal	Ben vs. Mal	Ben vs. Mal
Marker	LP	CA125	LP	CA125	HE4	ROMA
n	31	31	40	40	40	40
Threshold	28.246	16.400	41.180	97.000	170.585	55.743
AUC [corrected]	0.973 [0.971]	0.845	1.000 [0.998]	0.880	0.765	0.840
CI low	0.909	0.696	1.000	0.765	0.592	0.699
CI high	1.000	0.978	1.000	0.963	0.912	0.962
Sens. [corrected]	0.950 [0.924]	0.750	1.000 [0.972]	0.900	0.700	0.800
Spec. [corrected]	1.000 [0.994]	1.000	1.000 [0.992]	0.700	0.950	0.900
PPV	1.000	1.000	1.000	0.750	0.933	0.889
NPV	0.917	0.688	1.000	0.875	0.760	0.818
Acc. [corrected]	0.968 [0.949]	0.839	1.000 [0.982]	0.800	0.825	0.850
DeLong *p*-value vs. LP		0.089		0.033	0.002	0.013

Performance metrics are reported at the Youden-optimized thresholds for each binary comparison, LP, linear predictor; Threshold, Youden-optimized threshold; AUC, area under the curve; CI low and CI high, lower and upper bounds of 95% bootstrap confidence interval; Sens. Sensitivity; Spec, specificity; PPV. Positive predictive value; NPV negative predictive value; Acc. Accuracy CA125, cancer antigen 125; HE4, human epididymis protein 4; ROMA, Risk of Ovarian Malignancy Algorithm. Values in brackets denote optimism-corrected estimates obtained by Harrell’s bootstrap procedure. DeLong *p*-values compare each comparator’s apparent AUC against the LP’s apparent AUC at the corresponding diagnostic boundary; AUCs were compared on the apparent values by construction, as optimism-corrected estimates lack the covariance structure required for DeLong’s test.

## Data Availability

The data presented in this study are available on request from corresponding authors, upon reasonable request.

## Supplementary Materials

The following supporting information can be downloaded at https://www.mdpi.com/article/10.3390/diagnostics16152365/s1, Figure S1: Plasma concentrations of the full panel across diagnostic categories; Table S1: Clinicopathological characteristics of the reduced cohort.

## Abbreviations

IARC	International Agency for Research on Cancer
OC	Ovarian Cancer
CA125	Cancer Antigen 125
EOC	Epithelial Ovarian Cancer
HE4	Human Epididymal Protein 4
ROMA	Risk of Ovarian Malignancy Algorithm
TGF	Transforming Growth Factor
IL	Interleukin
CMNO	West Medical Center
CMIA	Chemiluminescent Microparticle Immunoassay
ELISA	Enzyme-Linked Immunosorbent Assay
TNF-α	Tumor Necrosis Factor-alpha
IFN-α	Interferon-alpha
IFN-γ	Interferon-gamma
FIGO	International Federation of Gynecology and Obstetrics
PPV	Positive Predictive Value
NPV	Negative Predictive Value
LP	Linear Predictor
AUC	Area Under the Curve
OR	Odds Ratio
CI	Confidence Interval
BH	Benjamini–Hochberg
FDR	False Discovery Rate
KW	Kruskal–Wallis
MW	Mann–Whitney
η^2^	Rank-based eta-squared effect size

## References

[1] Mazidimoradi A., Momenimovahed Z., Allahqoli L., Tiznobaik A., Hajinasab N., Salehiniya H., Alkatout I. (2022). The global, regional and national epidemiology, incidence, mortality, and burden of ovarian cancer. Health Sci. Rep..

[2] Ferlay J., Colombet M., Soerjomataram I., Parkin D.M., Pineros M., Znaor A., Bray F. (2021). Cancer statistics for the year 2020: An overview. Int. J. Cancer.

[3] Ma X., Qu L., Wu Q., Yu Q., Lin Z., Yang Y., Zhou X. (2025). Advances in ovarian cancer: Biological insights, therapeutic innovations, and future perspectives. J. Ovarian Res..

[4] Crum C.P., Drapkin R., Miron A., Ince T.A., Muto M., Kindelberger D.W., Lee Y. (2007). The distal fallopian tube: A new model for pelvic serous carcinogenesis. Curr. Opin. Obs. Gynecol..

[5] Momenimovahed Z., Tiznobaik A., Taheri S., Salehiniya H. (2019). Ovarian cancer in the world: Epidemiology and risk factors. Int. J. Womens Health.

[6] Siegel R.L., Giaquinto A.N., Jemal A. (2024). Cancer statistics, 2024. CA Cancer J. Clin..

[7] Fahmi M.N., Pradjatmo H., Astuti I., Nindrea R.D. (2021). Cytokines as Prognostic Biomarkers of Epithelial Ovarian Cancer (EOC): A Systematic Review and Meta-Analysis. Asian Pac. J. Cancer Prev..

[8] Terlikowska K.M., Dobrzycka B., Witkowska A.M., Mackowiak-Matejczyk B., Sledziewski T.K., Kinalski M., Terlikowski S.J. (2016). Preoperative HE4, CA125 and ROMA in the differential diagnosis of benign and malignant adnexal masses. J. Ovarian Res..

[9] Zhang R., Siu M.K.Y., Ngan H.Y.S., Chan K.K.L. (2022). Molecular Biomarkers for the Early Detection of Ovarian Cancer. Int. J. Mol. Sci..

[10] Cândido E.B., Silva L.M., Carvalho A.T., Lamaita R.M., Filho R.M.P., Cota B.D.C.V., da Silva-Filho A.L. (2013). Immune Response Evaluation Through Determination of Type 1, Type 2, and Type 17 Patterns in Patients With Epithelial Ovarian Cancer. Reprod. Sci..

[11] Govindaraj C., Scalzo-Inguanti K., Madondo M., Hallo J., Flanagan K., Quinn M., Plebanski M. (2013). Impaired Th1 immunity in ovarian cancer patients is mediated by TNFR2+ Tregs within the tumor microenvironment. Clin. Immunol..

[12] Hourani T., Sharma A., Luwor R.B., Achuthan A.A. (2025). Transforming growth factor-β in tumor microenvironment: Understanding its impact on monocytes and macrophages for its targeting. Int. Rev. Immunol..

[13] Alsina-Sanchís E., Figueras A., Lahiguera A., Gil-Martín M., Pardo B., Piulats J.M., Martí L., Ponce J., Matias-Guiu X., Vidal A. (2017). TGFβ Controls Ovarian Cancer Cell Proliferation. Int. J. Mol. Sci..

[14] Do T.V., Kubba L.A., Du H., Sturgis C.D., Woodruff T.K. (2008). Transforming growth factor-beta1, transforming growth factor-beta2, and transforming growth factor-beta3 enhance ovarian cancer metastatic potential by inducing a Smad3-dependent epithelial-to-mesenchymal transition. Mol. Cancer Res..

[15] Zhou J., Jiang W., Huang W., Ye M., Zhu X. (2020). Prognostic Values of Transforming Growth Factor-Beta Subtypes in Ovarian Cancer. BioMed Res. Int..

[16] Kumar P., Ranmale S., Mehta S., Tongaonkar H., Patel V., Singh A.K., Mania-Pramanik J. (2023). Immune profile of primary and recurrent epithelial ovarian cancer cases indicates immune suppression, a major cause of progression and relapse of ovarian cancer. J. Ovarian Res..

[17] Habel A., Xu W., Hadj Ahmed M., Stayoussef M., Bouaziz H., Ayadi M., Mezlini A., Larbi A., Yaacoubi-Loueslati B. (2023). Identification of two theranostic biomarker panels for epithelial ovarian cancer. Cytokine.

[18] Zheng H., Guan X., Meng X., Tong Y., Wang Y., Xie S., Guo L., Lu R. (2023). IFN-γ in ovarian tumor microenvironment upregulates HLA-E expression and predicts a poor prognosis. J. Ovarian Res..

[19] Pawlik W., Pawlik J., Kozłowski M., Łuczkowska K., Kwiatkowski S., Kwiatkowska E., Machaliński B., Cymbaluk-Płoska A. (2021). The Clinical Importance of IL-6, IL-8, and TNF-α in Patients with Ovarian Carcinoma and Benign Cystic Lesions. Diagnostics.

[20] Dobrzycka B., Terlikowski S.J., Garbowicz M., Niklińska W., Bernaczyk P.S., Nikliński J., Kinalski M., Chyczewski L. (2009). Tumor necrosis factor-alpha and its receptors in epithelial ovarian cancer. Folia Histochem. Cytobiol..

[21] Harrell F.E. (2015). Regression Modeling Strategies: With Applications to Linear Models, Logistic and Ordinal Regression, and Survival Analysis.

[22] DeLong E.R., DeLong D.M., Clarke-Pearson D.L. (1988). Comparing the areas under two or more correlated receiver operating characteristic curves: A nonparametric approach. Biometrics.

[23] Gao J., Zhu Y., Nilsson M., Sundfeldt K. (2014). TGF-β isoforms induce EMT independent migration of ovarian cancer cells. Cancer Cell Int..

[24] Wu Y.-H., Huang Y.-F., Chang T.-H., Chen C.-C., Wu P.-Y., Huang S.-C., Chou C.-Y. (2021). COL11A1 activates cancer-associated fibroblasts by modulating TGF-β3 through the NF-κB/IGFBP2 axis in ovarian cancer cells. Oncogene.

[25] Schauer I.G., Sood A.K., Mok S., Liu J. (2011). Cancer-associated fibroblasts and their putative role in potentiating the initiation and development of epithelial ovarian cancer. Neoplasia.

[26] Kioi M., Kawakami M., Shimamura T., Husain S.R., Puri R.K. (2006). Interleukin-13 receptor α2 chain. Cancer.

[27] Fujisawa T., Joshi B.H., Puri R.K. (2012). IL-13 regulates cancer invasion and metastasis through IL-13Rα2 via ERK/AP-1 pathway in mouse model of human ovarian cancer. Int. J. Cancer.

[28] Ripley D., Shoup B., Majewski A., Chegini N. (2004). Differential expression of interleukins IL-13 and IL-15 in normal ovarian tissue and ovarian carcinomas. Gynecol. Oncol..

[29] Moradi M.M., Carson L.F., Weinberg B., Haney A.F., Twiggs L.B., Ramakrishnan S. (1993). Serum and ascitic fluid levels of interleukin-1, interleukin-6, and tumor necrosis factor-alpha in patients with ovarian epithelial cancer. Cancer.

[30] Amer H., Kartikasari A.E.R., Plebanski M. (2021). Elevated Interleukin-6 Levels in the Circulation and Peritoneal Fluid of Patients with Ovarian Cancer as a Potential Diagnostic Biomarker: A Systematic Review and Meta-Analysis. J. Pers. Med..

[31] Liu C., Wang D., Huang X., Song Z., Ye L., Zhou G. (2025). The expression and clinical significance of cytokines Th1, Th2, and Th17 in ovarian cancer. Am. J. Med. Sci..

[32] Mollaoglu G., Tepper A., Falcomatà C., Potak H.T., Pia L., Amabile A., Mateus-Tique J., Rabinovich N., Park M.D., LaMarche N.M. (2024). Ovarian cancer-derived IL-4 promotes immunotherapy resistance. Cell.

[33] Santos T.D., Jammal M.P., Silveira T.P., Murta E.F.C., Nomelini R.S. (2019). Stromal IL2 is related to the neutrophil/lymphocyte ratio in epithelial ovarian cancer. Pathologica.

[34] Mielczarek-Palacz A., Kruszniewska-Rajs C., Smycz-Kubańska M., Strzelczyk J., Szanecki W., Witek A., Gola J.M. (2021). The Assessment of IL-21 and IL-22 at the mRNA Level in Tumor Tissue and Protein Concentration in Serum and Peritoneal Fluid in Patients with Ovarian Cancer. J. Clin. Med..

[35] Winkler I., Woś J., Karczmarczyk A., Miotła P., Gogacz M., Skorupska K., Rechberger T., Tabarkiewicz J., Wolińska E., Skrzypczak M. (2020). An association of circulating Tregs and Th17 cells producing IL-21 and IL-22 with the ROMA in ovarian cancer patients. Cytokine.

[36] Liu Z., Wu J., Wang X., Ji X. (2022). Multivariate logistic regression analysis of the correlation between five biomarkers and ovarian cancer in patients with intermediate-risk: A prospective cross-sectional study. Front. Cell Dev. Biol..

[37] Winarno G.N.A., Harsono A.B., Suardi D., Salima S., Mantilidewi K.I., Bayuaji H., Mulyantari A.I., Yulianto F.A., Susiarno H. (2024). Nomogram development for predicting ovarian tumor malignancy using inflammatory biomarker and CA-125. Sci. Rep..

[38] Moore R.G., Blackman A., Miller M.C., Robison K., DiSilvestro P.A., Eklund E.E., Strongin R., Messerlian G. (2019). Multiple biomarker algorithms to predict epithelial ovarian cancer in women with a pelvic mass: Can additional makers improve performance?. Gynecol. Oncol..

[39] Molina-Pineda A., Jauregui-Salazar F.O., Zamudio-Martínez A.G., Vizcarra-Ramos S., García-Gómez J., González-Amézquita B., Aguilar-Vazquez L.M., Villegas-Pacheco R., Hernandez-Gutierrez R., Jave-Suárez L.F. (2025). Utility of a Multimodal Biomarker Panel and Serum Proapoptotic Activity to Refine Diagnosis of Ovarian Adnexal Masses. Diseases.

