# Supplementary material for: Discovery and Development of a TGFβ3/IL-13 Plasma Risk Score for Preoperative Discrimination of Benign and Malignant Ovarian Adnexal Masses
Source: Diagnostics (Basel). 2026 Jul 28;16(15):2365. doi: 10.3390/diagnostics16152365 (PMC13464865; doi:10.3390/diagnostics16152365)
Supplement: Supplementary file 1 [file diagnostics-16-02365-s001.zip › Table S1.pdf]

Supplementary Table S1. Clinicopathological characteristics of the reduced cohort.

Characteristic	Control	Benign	Malignant	Test	p (raw)	q (BH-adj)
n	11	20	20			
Age, years. Median [IQR]	47.0 [43.0–51.5]	43.5 [40.0–51.5]	51.5 [44.8–56.2]	KW	0.107	0.154
Age at menarche, years. Median [IQR]	12.0 [11.0–12.5]	13.0 [11.8–13.2]	12.0 [11.0–13.0]	KW	0.228	0.270
Parity. Median [IQR]	2.0 [1.0–2.0]	2.0 [0.0–3.0]	1.0 [1.0–3.0]	KW	0.802	0.802
Nulliparous, n (%)	0 (0.0%)	5 (25.0%)	4 (20.0%)	χ^2	0.204	0.265
Postmenopausal, n (%)	8 (72.7%)	6 (30.0%)	13 (65.0%)	χ^2	0.028	0.046
Age at menopause, years. Median [IQR]	42.0 [41.0–42.2]	51.0 [48.5–53.5]	48.0 [40.8–49.2]	KW	0.011	0.020
CA125, U/mL. Median [IQR]	9.0 [7.5–10.0]	33.1 [15.1–137.7]	362.0 [116.2–1074.0]	KW	<0.001	<0.001
HE4, pmol/L. Median [IQR]	–	106.9 [81.0–126.0]	198.9 [146.8–223.1]	MW	0.004	0.009
ROMA, %. Median [IQR]	–	27.1 [19.7–39.4]	72.8 [60.4–89.0]	MW	<0.001	0.001
Largest tumour diameter, cm. Median [IQR]	–	7.5 [6.3–9.8]	11.9 [8.2–17.2]	MW	0.003	0.009
Bilateral, n (%)	–	2 (10.0%)	5 (25.0%)	Fisher	0.407	0.441
Histological subtype, n (%)						
Endometrioma / endometriosis	–	10 (50.0%)	–			
Serous cystadenoma	–	5 (25.0%)	–			
Mucinous cystadenoma	–	3 (15.0%)	–			
Other benign	–	2 (10.0%)	–			
High-grade serous	–	–	6 (30.0%)			
Low-grade serous	–	–	1 (5.0%)			
Endometrioid	–	–	3 (15.0%)			
Clear cell	–	–	3 (15.0%)			
Mucinous	–	–	5 (25.0%)			
Other malignant	–	–	2 (10.0%)			
FIGO stage (malignant), n (%)						
Stage I	–	–	7 (35.0%)			
Stage II	–	–	2 (10.0%)			
Stage III	–	–	9 (45.0%)			
Stage IV	–	–	1 (5.0%)			
Unspecified	–	–	1 (5.0%)			
Tumor grade (malignant), n (%)						
High grade	–	–	9 (45.0%)			
Low grade	–	–	1 (5.0%)			
Grade not specified	–	–	10 (50.0%)			

Continuous variables are reported as median [interquartile range, IQR]; categorical variables as n (%). Intergroup comparisons were performed using the Kruskal-Wallis test (KW) for continuous variables, the chi-squared test (χ^2) or Fisher's exact test for categorical variables, and the Mann-Whitney U test (MW) for variables available in two groups only. p (raw), unadjusted p-value; q (BH-adj), Benjamini-Hochberg adjusted q-value; ROMA, Risk of Ovarian Malignancy Algorithm; FIGO, International Federation of

Gynecology and Obstetrics; CA125, cancer antigen 125; HE4, human epididymis protein 4; ROMA, Risk of Ovarian Malignancy Algorithm. Dashes indicate variables not applicable in that particular group. Women with adnexal masses were enrolled on the basis of a preoperatively identified adnexal mass on transvaginal ultrasonography and confirmed histologically at surgery. Control volunteers had no adnexal pathology confirmed by transvaginal ultrasonography and CA125 measurement. HE4 and ROMA were available for participants with adnexal masses only and are therefore not reported for control volunteers. Dashes (—) indicate variables not applicable in that particular group. Other benign (n=2) comprises one leiomyoma and one case of ovarian stroma with cystic degeneration, both of mesenchymal origin. Other malignant (n=2) comprises one adenocarcinoma not otherwise specified (epithelial origin) and one adult granulosa cell tumor (sex-cord/stromal origin).
